# Treatment of Diabetic Gastroparesis by Complementary and Alternative Medicines

**DOI:** 10.3390/medicines2030212

**Published:** 2015-08-04

**Authors:** Hao Liu, Bo Yu, Meng Zhang, Kun Liu, Fu-Chun Wang, Xin-Yan Gao

**Affiliations:** 1 Institute of Acupuncture and Moxibustion, China Academy of Chinese Medical Sciences, 16 Nanxiaojie, Dongzhimennei, Beijing 100700, China; E-Mails: tommy_haozi@163.com (H.L.); zhangmeng7513@126.com (M.Z.); liukun0125@163.com (K.L.); 2 School of Acupuncture and Tuina-massage, Changchun University of Traditional Chinese Medicine, Changchun 130117, China; E-Mail: yubobj@163.com

**Keywords:** complementary and alternative medicine, diabetic gastroparesis

## Abstract

Gastroparesis is a common gastrointestinal complication in diabetes, induced by hyperglycemia and characterized by delayed gastric emptying and upper abdominal symptoms, such asnausea, vomiting, early satiety, bloating and epigastric pain. Diabetic gastroparesis (DGP) affects life quality and glycemic control, and is a challenge to treat in both Western and Eastern medicine. Routine treatment in Western medicine includes gastric emptying promoted by prokinetic agents, gastric pacemaking, or surgery combined with lifetime hormono-therapy, all of which have unavoidable side effects and limitations, and are very expensive. Complementary and alternative medical treatments like acupuncture, moxibustion, and massage are becoming more and more attractive because of their effectiveness, fewer side effects, and reliable safety. This article aims to introduce representative methods of complementary and alternative medicine to treat DGP, which were searched in English through Pubmed and in Chinese through CNKI (China Knowledge Resource Integrated Database). Several lines of evidence demonstrated the effects of single or combined complementary alternative therapies on DGP outcomes; however, the mechanisms were rarely investigated. Randomized controlled trials are undoubtedly required in future studies.

## 1. Introduction

Diabetic gastroparesis (DGP) is a type of chronic diabetic complication characterized by delayed gastric emptying and upper abdominal symptoms, such as nausea, vomiting, early satiety, bloating and epigastric pain [1]. A 10-year community-based study of gastroparesis in Olmsted County showed that there was a cumulative gastroparesis incidence of 5.2% and 1.0% in Type1 and Type 2 diabetics, respectively, which was much higher than 0.2% incidence in non-diabetic people [2]. Their life quality and blood glucose control maybe influenced by delayed gastric emptying, gastric dysrhythmia, pylorus spasm, non-synchronized gastroduodenal motility, and impaired pool meal accommodation because these symptoms are known to induce malnutrition and serious glucose variability [3,4]. Routine Western medicine therapy for gastroparesis includes prokinetic agents, gastric pacemaking, and surgery. However, side effects like extrapyramidal symptoms, cardiac arhythmia, local infection, malnutrition, and weight loss are inevitable [5]. Acupuncture, moxibustion and massage are complementary alternative medicine techniques with reliable efficacy and fewer side effects, and are quickly becoming more widely used world-wide. Many reports have indicated that complementary alternative medicine can improve gastrointestinal motility and accelerate gastric emptying, suggesting a possible treatment approach for DGP. Here, we report the different representative methods of complementary and alternative medicines in DGP treatments, as discovered through Pubmed and CNKI in English and Chinese publications, respectively.

## 2. Acupuncture Treatment

Zhang *et al* conducted a multicenter, randomized, controlled trial utilizing 200 patients with or without DGP [6]. The effect of acupuncture was compared to that of domperidone by observing patients’ symptom scales. The clinical efficacy rate in the acupuncture-treated group was 86.73%, significantly higher than that of the domperidone-treated control group. Acupuncture treatment exhibited a more significant effect on improving anorexia, belching, nausea, vomiting and epigastic pain compared to domperidone, and produced a more significant improvement in early satiety and bloating. Additionally, acupuncture was overall safer and produced fewer side effects than domperidone treatment.

Kim *et al* reported one DGP patient receiving acupuncture while continuing dynamic drug treatment [7]. After eight weeks of acupuncture treatment, the total gastroparesis cardinal symptom index score (range 0–5) reduced from 2.4 to 0.6, and gastric emptying time of solid foods decreased from 135 to 93 min. The four-month follow-up examination reported complete healing of subjective symptoms, but gastric emptying scanning was not reexamined as an objective evaluation.

Zheng *et al* conducted a trial, including 62 DGP patients to compare the effects of acupuncture at Ashi-point and conventional acupoints [8]. The Ashi-point was located via X-ray guidance, at the upper 1/3 and middle 1/3 junction of the greater curvature along the stomach. The positive and negative electrodes of the electroacupuncture (EA) were put on Zhongwan (RN12) and Ashi-point, respectively. Both acupoint assignments produced curative properties, as displayed by gastrointestinal barium meal scanning detection.

Abdominal acupuncture is a branch of acupuncture therapy in which acupoints around the umbilicus are selected for acupuncture stimulation. In a pilot study involving 60 DGP patients, the abdominal acupuncture group showed a greater serum concentration of motilin compared to the mosapride-treated group [9]. Furthermore, a significant decrease of glycosylated hemoglobin was found in the acupuncture-treated group, but not in the mosapride-treated group, which may suggest that abdominal acupuncture treatment is more effective than mosapride in regulating blood glucose during the treatment in weeks of time. Glycosylated hemoglobin is an irreversible biomarker for diabetes and it appears three months later than hyperglycaemia in the patient’s blood. Acupuncture might improved inner physicochemical environment by control the blood glucose to regulate DGP by maintain a homeostasis.

Xue *et al* observed the effect of acupuncture using an elongated needle (Mang needle) on gastrointestinal symptoms and blood glucose compared to domperidone administration in 85 DGP patients [10]. The total effectiveness rate of acupuncture treatment (86.7%) was significantly higher than that of domperidone treatment (55%), and blood glucose decreased more significantly in the acupuncture-treated group. Others addressed that the elongated needle on RN12 may stimulate celiac plexus and branches of vagus nerve to modulate the gastrointestinal functions [11].

Tang *et al* screened 10 clinical research articles (627 cases) about acupuncture treatment of DGP out of 37 reports. Most of the articles used randomized, controlled methods [12]. Only one trial employed single-blinded methods. One study performed a six months’ follow-up. Domperidone was used as a control treatment in eight trials, and cimetidine and mosapride were also selected as control treatments in other studies. Acupuncture-treated patients reported no adverse reactions, whereas drug-treated patients did report adverse side-effects in two trials.

Another systematic review indicated that acupuncture produced significant success on improving upper abdominal syndromes, but not on gastric emptying. Conditions of follow-up and long-term prognoses of those trials are unknown [13].

## 3. Massage Treatment

Liu *et al* compared the effect of abdominal massage to oral mosapride treatment in 108 DGP patients [14]. Abdominal massage improved anorexia, bloating, and epigastric pain better than mosapride treatment, but failed to show a significant difference in improving belching, nausea and vomiting.

In another study including 100 DGP patients, acupoint massage (on ST36 and RN12) combined with domperidone treatment exhibited amore distinct effect on decreasing plasma glucose and facilitating gastric emptying than that of domperidone alone [15].

## 4. Integrated Complementary and Alternative Medicine

Electroacupuncture (EA) combined with diet therapy was examinedin 60 cases of DGP [16]. After treatment, gastric hypomotility (delayed gastric emptying) was improved more significantly in the integrated acupuncture group than that in a control group of patients treated with domperidone combined with diet management. The total effectiveness rate of the integrated acupuncture group was 97%, which was significantly more than that of the control domperidone group (87%).

Li *et al* compared the efficiencies of acupuncture combined with traditional Chinese medicine (TCM) against mosapride treatment on symptoms and gastric emptying in 60 patients with DGP. Stimulating acupoints at the upper abdomen and forearm had a better effect on improving symptoms and delayed gastric emptying than mosapride treatment. Otherwise, serum somatostatin, which is produced by gastric parietal D cells and induces motility inhibitory, was decreased in the acupuncture/TCM treatment group, but not in the mosapride group [17].

In a trial of 64 DGP patients, the combination of acupuncture and auricular acupoint pressure was more effective on the epigastric symptoms bloating, belching, and acid reflux than mosapride treatment alone. However, both treatments exhibited similar effects on anorexia and epigastric pain reduction [18].

Moxibustion is usually used for cold or hypofunction syndromes in TCM. Fifty inpatients were divided into either warm acupuncture combined with indirect monkshood moxibustion treatment or mosapride treatment groups. Both groups displayed decreased plasma motilin after treatment. The total effectiveness rate of integrated moxibustion and acupuncture treatment (92%) was higher than that of mosapride treatment (72%). Motilin is a kind of gastrointestinal hormone always found with high level in DGP patient’s plasma. Acupuncture and moxibustion reduced the compensatory motilin release and consequently improved gastric motility [19].

Another systematic review including a meta-analysis [20] evaluated the efficacy of manual acupuncture, EA, warm acupuncture, acupuncture combined with acupoint massage and chiropractic for 14 randomized controlled trials of DGP. This analysis found that complementary alternative medicine was more effective in improving dyspeptic symptoms like nausea, vomiting, early satiety, bloating and epigastric pain. Complementary alternative medicines were found to have equal efficacy as prokinetics. No adverse reactions occurred in alternative medicine treatment groups, whereas some mild side effects were found in some control groups.

## 5. Experimental Studies

We recently reported the effect of acupuncture at 16 acupoints with different segmental locations on gastric motility in anesthetized rats. We found that acupuncture at Sibai (ST2), Juliao (ST3), an auricular point on the head, Quchi (LI11) on the forelimb, Qihu (ST13) on the upper thorax, Feishu (BL13) on the upper back, Xiaochangshu (BL27) on the lower back, Futu (ST32), Zusanli (ST36), Sanyinjiao (SP6) on the hindlimb, and a point on the tail, which were all in heterotopic spinal segment innervations to the stomach, produced a significant excitatory effect on gastric motility.Conversely, the same stimulation at Rugen (ST18) on the lower thorax, Zhongwan (CV12), Liangmen (ST21), Qihai (CV6) on the abdomen, and Weishu (BL21) on the middle back, which are in homotopic spinal innervations with the stomach, inhibited gastric motility [21].

Our study demonstrated that acupuncture modulates gastric motility via the autonomic nervous system [22]. When acupuncture was performed at ST21 or CV6 on the abdomen, the activity of sympathetic nerve’s gastric branch was excited whereas the vagus nerve of the gastric branches was slightly dampened or unchanged. However, opposite changes of autonomic nervous activity were observed when LI11 and ST36 on the limbs were stimulated. Our unpublished data in normal and DGP model rats were consistent with the previous report, never the less, the nerve activities were sparse due to hyperglycemia in the DGP model rats. Acupuncture at single or combined heterotopic acupoints increased the amplitude of gastric motility and the discharge frequency of the subdiaphragamatic vagus nerve. Acupuncture at homotopic acupoints inhibited gastric motility but excited postganglionic efferent of the celiac ganglion. Homotopic acupoint stimulation exerted a dominate effect on gastric function when combined with heterotopic acupoint stimulation.

Su *et al* observed the effects of different acupoint stimulation on gastric motility in acid-sensing ion channel 3 knockout (ASIC3^−/−^) mice, transient receptor potential vanilloid receptor 1 knockout (TRPV1^−/−^) mice, and C57BL/6 mice as a wild type control. The results showed that EA at an acupoint on the hind limbs (ST36) increased intragastric pressure and peristole amplitude while EA at an abdominal acupoint (RN12) inhibited these effects in all of the three animal groups. Excitatory and inhibitory effects of EA on gastric motility in TRPV-1^−/−^ mice were significantly decreased compared with C57BL/6, whilethe decrease was minimalin ASIC3^−/−^ mice.

Regarding stimulation intensity, EA at ST36 could promote gastric motility if the stimulation intensity was at the Aδ-/C-afferent fiber threshold (TAδ/TC). The effect of EA increased with the increase of stimulation intensity in the range of TAδ and TC, however, the modulatory effect of EA on gastric motility could be saturated when the stimulation intensity exceeded TC [23].

## 6. Conclusions

Complementary alternative medicine has been widely used to treat functional gastrointestinal diseases such as gastroparesis, dyspepsia, diarrhea, constipation, and irritable bowel syndrome [24]. Gastroparesis is a common gastrointestinal complication in diabetes, characterized by delayed gastric emptying and various upper abdominal symptoms. Previous reports have demonstrated that complementary therapy could significantly improve epigastric symptoms and gastric emptying in gastroparesis [19,25,26]. Animal experiments revealed that acupuncture could modulate gastric motility via the autonomic nervous system in normal and diabetic model rats [27,28,29]. Various complementary therapeutics exhibited possibilities for the treatment of diabetic gastroparesis. PC6, RN12, ST36 are the most frequently selected acupoints when treating gastric disorders. Acupuncture at heterotopic acupoints could improve gastroparesis via excitation of vagal nerves and inhibition of sympathetic nerves. Future studies should focus on optimized treatment plans rather than efficacy verification. Additionally, the biological mechanism of acupoints used in combination should be considered. Overall, complementary and alternative medicine as affordable and safe DGP treatment options show promising future prospects. More well-designed, larger-scale, and long-term follow-up trials regarding complementary and alternative medicinesare needed to provide stronger evidence to support their utility as a favorable DGP treatment.
